# A minimal power model for human running performance

**DOI:** 10.1371/journal.pone.0206645

**Published:** 2018-11-16

**Authors:** Matthew Mulligan, Guillaume Adam, Thorsten Emig

**Affiliations:** 1 Claremont McKenna College, W.M. Keck Science Department, Claremont, California, United States of America; 2 Massachusetts Institute of Technology, MultiScale Materials Science for Energy and Environment, Joint MIT-CNRS Laboratory (UMI 3466), Cambridge, Massachusetts, United States of America; 3 Laboratoire de Physique Théorique et Modèles Statistiques, CNRS UMR 8626, Bât. 100, Université Paris-Saclay, Orsay cedex, France; National Institutes of Health, National institute of Diabetes and Digestive and Kidney Diseases, UNITED STATES

## Abstract

Models for human running performances of various complexities and underlying principles have been proposed, often combining data from world record performances and bio-energetic facts of human physiology. The purpose of this work is to develop a novel, minimal and universal model for human running performance that employs a relative metabolic power scale. The main component is a self-consistency relation for the time dependent maximal power output. The analytic approach presented here is the first to derive the observed logarithmic scaling between world (and other) record running speeds and times from basic principles of metabolic power supply. Our hypothesis is that various female and male record performances (world, national) and also personal best performances of individual runners for distances from 800m to the marathon are excellently described by this model. Indeed, we confirm this hypothesis with mean errors of (often much) less than 1%. The model defines endurance in a way that demonstrates symmetry between long and short racing events that are separated by a characteristic time scale comparable to the time over which a runner can sustain maximal oxygen uptake. As an application of our model, we derive personalized characteristic race speeds for different durations and distances.

## Introduction

Scientists have been fascinated by trying to explain running performance and to predict its limitations for more than 100 years. A purely descriptive approach was employed by Kennelly as early as 1906 for speeds in racing events of animals and humans. For men running events from 20 yards up to a few hundred miles he found a power law relation between distance *d* and duration *T* with *T* ∼ *d*^9/8^ with a relative large error of up to 9% for distances from 100m to 50 miles (and larger errors for shorter and longer distances) [1].

Almost a century ago, in 1925 noted mathematician and physiologist A.V. Hill proposed a power model based on metabolic energy considerations to describe the maximal power output *P*_*max*_(*T*) over a given duration *T* by a hyperbolic function *P*_*max*_(*T*) = *P*_0_ + *P*_1_/*T* with constants *P*_0_ and *P*_1_ (known as the “running curve”) [2]. Ward-Smith introduced a model, based on the first law of thermodynamics, to describe performances at Olympic Games from 1960 to 1976 with an average absolute error for the predicted times of 0.86% for distances from 100m to 10,000m [3]. In 1973 the mathematician Keller formulated a purely mechanical model that is based on the runner’s equation of motion with a damping term [4]. The propulsive force is connected to the mechanical power utilized for running which is different from the overall metabolic power requirement. In analogy to purely mechanical problems, Keller assumed that the damping is linear in velocity and that the damping coefficient is constant over time. The justification for these assumptions is not validated given that a comparison of his model to world track records from 50yards to 10,000m yields a relative large errors of about 3% for distances larger than 5000m. Furthermore, both Hill’s and Keller’s models predict the existence of a maximal speed that can be sustained for an infinite duration, which is not possible from a physiological point of view and incompatible with data on running records. Similarly, a threshold power has been proposed by Jones et al. in the critical power model [5].

In fact, existing models appear to be unable to *explain* an important observation that has been made already by Hill in the context of his above mentioned model: The average fractional utilization of maximal power (or the average running speed) of world record performances scales linearly with the *logarithm* of the duration of the performance [2]. An interesting model that interpolates between fundamental knowledge of human bioenergetics during exercise and actual world record running performance was proposed by Peronnet and Thibault [6, 7]. Their model combines characteristics of energy metabolism, based on Hill’s hyperbolic “running curve” and the dynamics of oxygen uptake. However, the fractional utilization of maximal power over a given duration is described in their model by a phenomenological logarithmic term that is based on observations in running records. The latter term accounts for endurance limited sustainability of maximal aerobic power. Currently, this model is most effective in reproducing world record running performances. However, it uses a number of fixed parameters that are assumed to be equal for all world record performances although they have been achieved by different athletes. In fact, many parameters can be different among individuals. For example, running economy, i.e., the energy cost of running at a given velocity, shows substantial inter-individual variation [8]. These variations are observed even among well trained elite runners. Another quantity that is modeled as a constant in Peronnet’s and Thibault’s model is the duration over which maximal aerobic power (or VO_2*max*_) can be maintained during running which they assumed to be 7 minutes. However, direct measurements of oxygen uptake have demonstrated variations of the order of one to two minutes among individuals [9, 10]. From a fundamental perspective it is desirable to derive a model from basic principles of metabolic power generation and utilization that predicts human performances without additional phenomenological input. This is the objective of the present work.

For the development of our model it is instructive to review some facts and experimental observations from exercise physiology. When developing a model that can describe running performances as obtained in world records up to the marathon distance one should realize at what relative intensities these races are performed. All Olympic endurance events require intensities above 85% of VO_2*max*_ which corresponds to the effort reached approximately in the marathon [11]. When looking at record performances, we can also assume that runner has followed an optimal carbohydrate loading strategy so that the stored amount of glycogen is permitting best possible performance. This is of importance for the half marathon and in particular the marathon distance which is raced predominantly on carbohydrate fuel with an average respiratory gas exchange ratio of close to one for faster runners [12].

An important physiological observation is that the total energy cost of running increases linearly with the covered distance with no or a very small dependence on the running velocity [13, 14]. Hence the power output changes linearly with speed, with the slope quantifying running economy. It is known that this running economy can vary about 30–40% among individuals [11]. An important observation that is essential for the construction of our model is that running economy usually becomes worse with the duration of a running event. The magnitude of the change in economy increases with duration and intensity. The actual change is probably subject dependent and also influenced by external conditions. We shall see below that this is an important factor in determining race velocities and endurance. This drift in running economy has been quantified in treadmill studies with a change of 4.4% for 40min at 80% VO_2*max*_, a change of 6.6% for 60min at 70% VO_2*max*_, and a change of 9.5% for 60min at 80% VO_2*max*_ [15]. An other study found for 60min treadmill running near 80% of VO_2*max*_ a shift of about 3% in oxygen uptake [16]. Changes in running economy have been also observed during a 5km run at a constant pace eliciting about 80–85% of VO_2*max*_ with an average increase in oxygen uptake of 3.3% for men and 2.0% for women [17]. The reason for the increase in oxygen uptake and reduction in running economy is unknown. A number of mechanisms have been postulated in the literature but most of them are speculative [12, 18–20], including an increase in oxygen uptake due to neuromuscular fatigue [21]. Without discussing here the various attempts that have been made for explaining this observation, we just conclude that every activated physiological system increases its own particular energy consumption with the duration of exercise.

## Methods

### A minimal model for running performance

In view of the current status of theoretical descriptions of human running performances, it appears useful to construct a *minimal and universal* model for human running performance that fulfills the following two requirements:

1. Based on basic concepts and observations on metabolic power generation and utilization during running
2. Minimal number of physiological parameters that are not fixed a priori

In order to eliminate irrelevant normalization parameters from the model (that would depend on the choice of units for energy, power, etc.), we express our model in terms of relative quantities. We shall base the model on expedited power measured as oxygen uptake per time since this quantity can be measured directly under real conditions by mobile spirometry. This implies a slight time dependence of oxygen uptake during prolonged exercise, even when the power output is constant, due to a change of the respiratory quotient with substrate utilization [22]. Also, since body weight usually changes during prolonged exercise, we measure power or oxygen uptake always per body weight.

While the basal metabolic rate *P*_*b*_ is close to 1.2W/kg [6], its actual value is not required in the following. In fact, in the parameterization of running economy to be employed below, we chose to associate *P*_*b*_ with the power that is obtained by linearly extrapolating the running economy to zero velocity. Hence we neglect the non-linear dependence of the energy cost on sub-running (walking) velocities which causes no problem since our model uses the energy cost of motion only in the linear running regime. In our model there exists a crossover power *P*_*m*_ that we expect to be close to the maximal aerobic power associated with maximal oxygen uptake VO_2*max*_ which is typically in the range of 75 to 85ml/(kg min) for elite runners [6]. The power *P*_*m*_ should not be confused with the critical or the maximal power that occurs in the 3-parameter critical power model of Morton [23].

We measure power relative to the base value *P*_*b*_, in units of the aerobic power reserve *P*_*m*_ − *P*_*b*_ that is available to the runner, hence defining the relative running power (or intensity) as

$$\begin{array}{c} p=\frac{P-P_{b}}{P_{m}-P_{b}} \end{array}$$ (1)

for a given power *P* so that 0 ≤ *p* ≤ 1 for running intensities that do not require more power than provided aerobically by maximal oxygen uptake.

Following the definition of the relative running power above, we parameterize the *nominal* power expenditure that is required to run at a velocity *v*, i.e., the running economy, as

$$\begin{array}{c} p\left(v\right)=\frac{P\left(v\right)-P_{b}}{P_{m}-P_{b}}=\frac{v}{v_{m}} \end{array}$$ (2)

where *v*_*m*_ is a crossover velocity which is the smallest velocity that elicits the nominal power *P*_*m*_. We expect this velocity to be close to the velocity that permits the runner to spent the longest time at maximal aerobic power [24]. Here “nominal” implies that this power is measured for short duration and idealized laboratory conditions under which running economy is linear in velocity, at least to a very good approximation [25]. For velocities *v* > *v*_*m*_ the energy cost of running cannot be determined from oxygen uptake measurements due to anaerobic involvement, and the actual (non-nominal) energy cost might increase in a non-linear fashion [12]. We shall see below that our model allows us to estimate this non-linear correction from the supplemental power required to race at a given velocity.

To model running performance, we need information on the maximal duration over which a runner can sustain a given power, and hence a certain running velocity. To quantify this information, we define *P*_*max*_(*T*) as the maximal *average* power that can be sustained over a duration *T*. This is the power (measured as oxygen uptake) that is *nominally* required to run at a given velocity. Hence *P*_*max*_(*T*) can be used to deduce the *mean* running velocity of an event of duration *T*. In addition, we define the instantaneous power *P*_*T*_(*t*) that a runner utilizes during a race (defined as an event in which a fixed distance is covered in minimal time) of duration *T* at time *t* with 0 ≤ *t* ≤ *T*. *P*_*T*_(*t*) should be regarded as “typical” power output at time *t* of an event of duration *T*, meaning that a given individual runner generates a power that in general fluctuates in time around *P*_*T*_(*t*). It is important to note that the instantaneous power *P*_*T*_(*t*) exceeds *P*_*max*_(*T*) due to an upward shift in the required power beyond the nominal power (for example due to decreased running economy, non-linear corrections for velocities above *v*_*m*_). The additional energy that is required to allow for this upward shift is assumed to grow linearly in time, providing an *supplemental power* *P*_*sup*_. We expect that this power is provided by different anaerobic and aerobic energy systems, involving different time scales over which they mainly contribute to *P*_*sup*_. Hence, we introduce a crossover time *t*_*c*_ that separates long (l) and short (s) running events, suggesting the parameterization

$$\begin{array}{c} P_{sup}\left(T\right)=P_{s}\;\;\text{for}\;T\leq t_{c}\;,\;\;P_{sup}\left(T\right)=P_{s}\frac{t_{c}}{T}+P_{l}\frac{T-t_{c}}{T}\;\;\text{for}\;T>t_{c}\;, \end{array}$$ (3)

which describes the fractional contribution of energy systems during short and long events of total duration *T*. While the sharp crossover between these regimes is an oversimplification of reality, we shall see below that it leads to reasonable estimates. We have assumed that there is only one crossover time scale since there exists only one distinct power scale *P*_*m*_ which is presumably set by the maximal aerobic power. Hence we associate *t*_*c*_ with the time scale over which maximal aerobic power can be sustained.

To construct our model, we start from the following self-consistency relation

$$\begin{array}{c} P_{max}\left(T\right)+P_{sup}\left(T\right)=\frac{1}{T}\int_{0}^{T}P_{T}\left(t\right)dt\;, \end{array}$$ (4)

which states that the sum of the nominal average power and the additional supplemental power *P*_*sup*_ equals the time average of the instantaneously utilized power. We make the important conjecture that the instantaneous power utilized at time *t* equals the maximal power that can be sustained for the remaining time *T* − *t* of the event [26], i.e.,

$$\begin{array}{c} P_{T}\left(t\right)=P_{max}\left(T-t\right)\;. \end{array}$$ (5)

Note that this implies that the power output during a race is not constant over time but increases towards the end of the event. When this relation is substituted into the self-consistency Eq (4), one obtains an integral equation that determines *P*_*max*_(*T*). If there would be no supplemental power (*P*_*sup*_ = 0) then the integral equation has a constant *P*_*max*_(*T*) as solution since *P*_*max*_(*T*) must be a non-increasing function of *T*. However, a constant solution is not acceptable since a given power cannot be sustained for all durations *T*, and hence *P*_*sup*_ must be non-zero. The general solution is (for details see S1 Appendix)

$$\begin{array}{c} P_{max}\left(T\right)=\{\begin{array}{cc} P_{m}-P_{s}\log\frac{T}{t_{c}} & \;\text{for}\;T\leq t_{c}\\ P_{m}-P_{l}\log\frac{T}{t_{c}} & \;\text{for}\;T>t_{c} \end{array}\;, \end{array}$$ (6)

where *P*_*m*_ = *P*_*max*_(*t*_*c*_) is the crossover power reached at the crossover time *t*_*c*_. We note that *P*_*max*_(*T*) can be compared to experimental studies of oxygen consumption during running for short durations below *t*_*c*_, see S2 Appendix.

It turns out to be useful to measure *P*_*s*_ and *P*_*l*_ as fractions of the aerobic power reserve *P*_*m*_ − *P*_*b*_ by introducing two corresponding dimensionless factors *γ*_*s*_ and *γ*_*l*_ that are defined by the relations

$$\begin{array}{c} \gamma_{s}=\frac{P_{s}}{P_{m}-P_{b}}\;,\;\;\gamma_{l}=\frac{P_{l}}{P_{m}-P_{b}}\;. \end{array}$$ (7)

This definition has the advantage that the duration *T* over which a runner can sustain a given power *P* can now be expressed as

$$\begin{array}{c} T\left(P\right)=\{\begin{array}{cc} t_{c}\exp[-\frac{1}{\gamma_{l}}\frac{P-P_{m}}{P_{m}-P_{b}}] & P\leq P_{m}\\ t_{c}\exp[-\frac{1}{\gamma_{s}}\frac{P-P_{m}}{P_{m}-P_{b}}] & P\geq P_{m} \end{array} \end{array}$$ (8)

or in terms of the relative power *p* [see Eq (1)] as

$$\begin{array}{c} T\left(p\right)=\{\begin{array}{cc} t_{c}\exp[-\frac{p-1}{\gamma_{l}}] & p\leq 1\\ t_{c}\exp[-\frac{p-1}{\gamma_{s}}] & p\geq 1 \end{array}\;. \end{array}$$ (9)

The time *T* over which an average velocity *v* can be sustained follows now directly by substituting the nominal running economy function of Eq (2) into the above equation, leading to

$$\begin{array}{c} T\left(v\right)=\{\begin{array}{cc} t_{c}\exp[\frac{v_{m}-v}{\gamma_{l}v_{m}}] & T\geq t_{c}\;\;\text{or}\;\;v\leq v_{m}\\ t_{c}\exp[\frac{v_{m}-v}{\gamma_{s}v_{m}}] & T\leq t_{c}\;\;\text{or}\;\;v\geq v_{m} \end{array}\;. \end{array}$$ (10)

The fastest performance time *T*(*d*) for a distance *d* can be obtained from Eq (10) by setting *v* = *d*/*T* and solving for *T*. The solution can be expressed as the real branch *W*_−1_(*z*) of the Lambert W-function which is defined as the (multivalued) inverse of the function *w* → *we*^*w*^ [27],

$$\begin{array}{c} T\left(d\right)=\{\begin{array}{cc} -\frac{d}{\gamma_{l}v_{m}}\frac{1}{W_{-1}[-\frac{d}{d_{c}\gamma_{l}}e^{-1/\gamma_{l}}]}\; & \;\text{for}\;\;d\geq d_{c}\\ -\frac{d}{\gamma_{s}v_{m}}\frac{1}{W_{-1}[-\frac{d}{d_{c}\gamma_{s}}e^{-1/\gamma_{s}}]}\; & \;\text{for}\;\;d\leq d_{c} \end{array}\;, \end{array}$$ (11)

where we have defined the distance *d*_*c*_ = *v*_*m*_*t*_*c*_. (The function *W*_−1_(*z*) is real valued for −1/*e* ≤ *z* < 0, a condition which is fulfilled for all distances *d* that we consider.) Note that *T*(*d*) is continuous at *d* = *d*_*c*_ with *T*(*d*_*c*_) = *t*_*c*_ since *W*_−1_(*we*^*w*^) = *w*.

This function *T*(*d*) can be used the estimate the model parameters *v*_*m*_, *t*_*c*_, *γ*_*l*_ and *γ*_*s*_ by minimizing the relative quadratic error between *T*(*d*_*j*_) and the actual race time over distance *d*_*j*_ for all races *j* = 1, …, *N*. We shall demonstrate this explicitly below. From the race time *T*(*d*) we can obtain the mean race velocity for a distance *d*, given by $\bar{v}\left(d\right)=d/T\left(d\right)$. When we express $\bar{v}\left(d\right)$ relative to *v*_*m*_, we obtain the expression

$$\begin{array}{c} \frac{\bar{v}\left(d\right)}{v_{m}}=\{\begin{array}{cc} -\gamma_{l}W_{-1}[-\frac{1}{\gamma_{l}}\frac{d}{d_{c}}e^{-1/\gamma_{l}}] & \;\text{for}\;\;d\geq d_{c}\\ -\gamma_{s}W_{-1}[-\frac{1}{\gamma_{s}}\frac{d}{d_{c}}e^{-1/\gamma_{s}}] & \;\text{for}\;\;d\leq d_{c} \end{array}\;, \end{array}$$ (12)

which depends only on the parameter *γ*_*l*_ (or *γ*_*s*_) in the long (or short) regime when the distance is measured in units of *d*_*c*_. This function will be shown below for world records and individual runners, and a typical range of values for *γ*_*l*_ and *γ*_*s*_.

In order to compare our model predictions to the often assumed power law or “broken power law” description of running records [1, 28], it is useful to perform an asymptotic expansion of the Lambert function *W*_−1_(*z*) for small negative *z*. This is justified since for all here considered distances *d* and model parameters, the argument of *W*_−1_ in Eq (11) never is smaller than −0.1. In this range a very good approximation (better than 0.4%) is given by $W_{-1}\left(z\right)=L_{1}\left(z\right)-L_{2}\left(z\right)+L_{2}\left(z\right)/L_{1}\left(z\right)+\left[L_{2}^{2}\left(z\right)-2L_{2}\left(z\right)\right]/\left[2L_{1}^{2}\left(z\right)\right]+\ldots$ with *L*_1_(*z*) = log(−*z*) and *L*_2_ = log(−log(−*z*)). Defining the re-scaled logarithmic time, distance and mean velocity variables *τ* = log(*T*/*t*_*c*_), *δ* = log(*d*/*d*_*c*_) and $\upsilon =\log(\bar{v}/v_{m})$, for *d* ≥ *d*_*c*_ the time-distance and velocity-distance relations are very well approximated by

$$\begin{array}{c} \tau \left(\delta \right)=\delta -\upsilon \left(\delta \right)\;,\;\upsilon \left(\delta \right)=L(\frac{1}{\gamma_{l}},\delta -\log\gamma_{l}) \end{array}$$ (13)

with

$$L\left(x,y\right)=-\log\left(x\right)+\log[x-y+\log\left(x-y\right)+\frac{\log(x-y)}{x-y}-\frac{\log\left(x-y\right)(\log(x-y)-2)}{2{\left(x-y\right)}^{2}}]\;.$$

The same relations hold for *d* ≤ *d*_*c*_ when *γ*_*l*_ is replaced by *γ*_*s*_ in Eq (13). Note that the relation between mean race velocity $\bar{v}$ and race distance *d* is not a power law as assumed in some studies [28, 29]. For example, Riegel’s formula corresponds in above notation to *τ*(*δ*) = *αδ* − *L*, *υ*(*δ*) = −(*α* − 1)*δ* + *L* with a *constant* *L* and an exponent *α* close to 1.06. Our model predicts that *α* = 1 exactly and that the very small deviation from *α* = 1, observed by Riegel and others, is due to a hierarchy of logarithmic corrections, giving rise to a non-constant *L*. It is interesting to observe from Eq (13) that the endurance measuring parameter *γ*_*l*_ or *γ*_*s*_ is the only quantity which determines the time to distance and velocity to distance relations when time is measured in units of *t*_*c*_ and velocity in units of *v*_*m*_. We note that for the comparison of our model to record performances and personal best performances of individual runners, we always use the exact expressions involving the Lambert W-function.

### Interpretation of supplemental power *P*_*sup*_, and of *γ*_*l*_, *γ*_*s*_

The supplemental power defined in Eq (3) can be expressed relative to the aerobic power reserve *P*_*m*_ − *P*_*b*_ as

$$\begin{array}{c} \frac{P_{sup}\left(T\right)}{P_{m}-P_{b}}=\gamma_{s}\;\;\text{for}\;T\leq t_{c}\;,\;\;\frac{P_{sup}\left(T\right)}{P_{m}-P_{b}}=\left(\gamma_{s}-\gamma_{l}\right)\frac{t_{c}}{T}+\gamma_{l}\;\;\text{for}\;T>t_{c}\;, \end{array}$$ (14)

where we used the definitions of Eq (7). The averaged utilized power during a race of duration *T* and mean velocity $\bar{v}\left(T\right)$, given by the inverse of Eq (10), is determined by the sum of nominal and supplemental power [see Eq (4)],

$$\begin{array}{c} P_{max}+P_{sup}=\{\begin{array}{cc} P_{b}+\frac{\bar{v}\left(T\right)}{v_{m}}[1+\frac{1}{1/\gamma_{s}-\log\left(T/t_{c}\right)}] & \;\text{for}\;\;T\leq t_{c}\\ P_{b}+\frac{\bar{v}\left(T\right)}{v_{m}}[1+\frac{1+\left(\gamma_{s}/\gamma_{l}-1\right)t_{c}/T}{1/\gamma_{l}-\log\left(T/t_{c}\right)}] & \;\text{for}\;\;T>t_{c} \end{array}\;. \end{array}$$ (15)

The factors in the square brackets measure the amount by which the total mean running power deviates from the nominal linear relation $P_{b}+\bar{v}\left(T\right)/v_{m}$ with increasing duration *T*. At the crossover time *t*_*c*_ the factor has its maximum with a value of 1 + *γ*_*s*_. Below, we shall show graphs of the duration dependence of these supplemental factors for running world records, and discuss them in relation to experimental observations.

### Endurance for short and long duration

**Fig 1:**
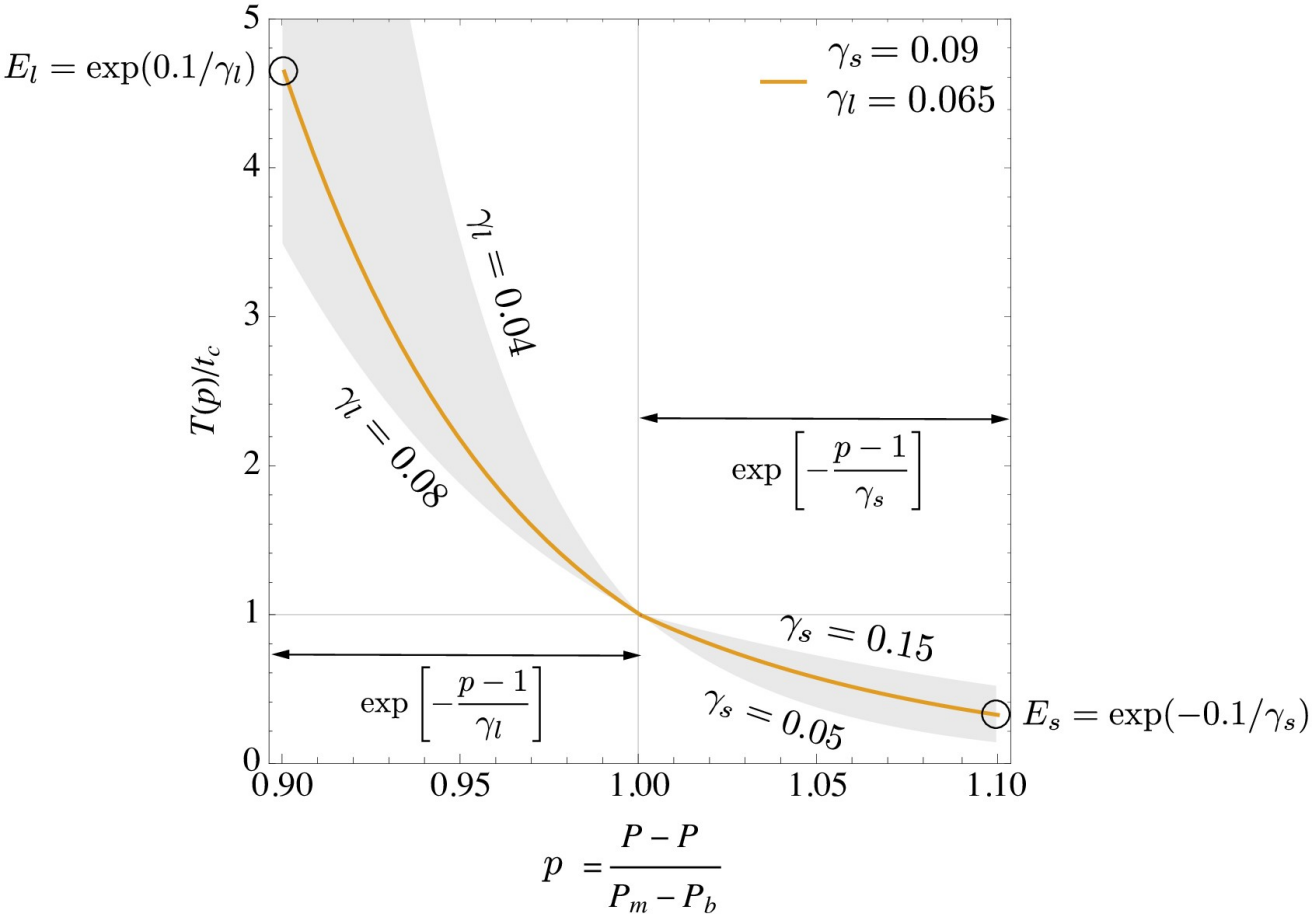
Definition of endurance for long and short duration, *E*_*l*_ and *E*_*s*_, respectively, from the duration *T*(*p*) over which a relative power *p* can be sustained. Shown is a typical range of endurances for long and short duration (gray regions, with lower and upper limits for *γ*_*l*_ and *γ*_*s*_) and an example curve that visualizes the definition of *E*_*l*_ and *E*_*s*_.

The duration *T*(*p*) over which a runner can sustain a given relative power *p* is shown in Fig 1 for typical values of the parameters *γ*_*l*_ and *γ*_*s*_. The long and short duration regimes are related by symmetry about the crossover point at *p* = 1 due to the same exponential increase (decease) of the duration *T*(*p*): Starting from the crossover power *P*_*m*_, corresponding to *p* = 1, the duration *T*(*p*) increases exponentially when the power output is reduced. The rate of this increase is controlled by the exponent *γ*_*l*_. Therefore we define an endurance for *long* duration as *E*_*l*_ = exp(0.1/*γ*_*l*_) so that the duration over which a runner can maintain 90% (*p* = 0.90) of crossover power is given by *T* = *t*_*c*_*E*_*l*_. Hence a smaller *γ*_*l*_ corresponds to better endurance. Similarly, one can ask what parameter range for *γ*_*s*_ yields a better performance on shorter distances below the crossover distance *d*_*c*_. Since in this short duration range one has *p* > 1, the exponential dependence of *T*(*p*) yields an increasing duration with increasing *γ*_*s*_. An endurance for *short* duration can hence be defined as *E*_*s*_ = exp(−0.1/*γ*_*s*_) so that a runner can sustain 110% of crossover power for a duration *T* = *t*_*c*_*E*_*s*_. Opposite to the long duration regime, here a larger *γ*_*s*_ corresponds to a better endurance. The choice of 90% and 110% of crossover power is arbitrary, and other sub- and supra-maximal values could be chosen to define endurances without any qualitative difference in interpretation. We shall come back to these endurance measures when we discuss personalized characteristic race paces.

### Estimation of physiological model parameters

Our model depends on the four independent parameters *v*_*m*_, *t*_*c*_, *γ*_*s*_ and *γ*_*l*_ that characterize a group of runners (for example world record holders) or individual runners. Otherwise our model is universal in the sense that it contains no additional fixed parameters or constants. The four parameters can be estimated from a given set of results (distance and time) from exercise performed at maximal intensity, i.e., races. These sets can be either records, like world records, involving a group of different runners or personal records (best performances) from individual runners. To check the accuracy of our model and to compute the model parameters, we minimize numerically the sum of the squared differences between the actual race time and the one predicted by Eq (11) for all results in a given set. This method will be used to reconstruct individual physiological profiles (running economy and endurance) from race performances in Application 1 below.

### Prediction of race times and characteristic paces for given times and distances

Once the model parameters for a given set of performance results have been determined, the model can be applied to compute a number of interesting quantities that could guide racing and training of a runner. For example, by comparing the time difference between the actual race time and the model’s prediction for all raced distances, preferred or optimal distances for a runner can be identified. For distances that have not been raced before, or only prior to a newly focused training program, the formula of Eq (11), or its approximative version in Eq (13), can be used to predict racing times.

Another application of our model is the estimation of characteristic velocities that correspond to a prescribed relative power output $\hat{p}$, measured in percent of aerobic power reserve that is available over a given duration. Generally, running velocities *v* in training units depend on the purpose of the training session and hence on duration *T* or distance *d* of the workout intervals. Suppose that a runner trains at a relative power $\hat{p}$. This relative power relates the target power output *P*(*v*) to the maximal power above basal power, *P*_*max*_(*T*) − *P*_*b*_, that can be maintained for the duration *T* by the relation

$$\begin{array}{c} P\left(v\right)=\hat{p}\left(P_{max}\left(T\right)-P_{b}\right)+P_{b}\;. \end{array}$$ (16)

Note that we define here the target power output not relative to the absolute crossover power but relative to the maximal aerobic power that can be sustained over time *T*. This is a natural choice since for a workout of duration *T*, the maximum power that can be maintained over that time is only *P*_*max*_(*T*). Let us assume that a runner would like to perform a continuous run over a time *T* at an intensity $\hat{p}$, e.g., at 90% ($\hat{p}=0.9$) of maximally possible intensity over that time *T*. Then Eq (16) determines under these conditions the velocity *v* for the run. An important observation is that the solution of Eq (16) is independent of both *P*_*b*_ and *P*_*m*_. In fact, it can be expressed as

$$\begin{array}{c} v\left(\hat{p},T\right)=\{\begin{array}{cc} \hat{p}\;v_{m}[1-\gamma_{l}\log\left(T/t_{c}\right)] & T\geq t_{c}\\ \hat{p}\;v_{m}[1-\gamma_{s}\log\left(T/t_{c}\right)] & T\leq t_{c} \end{array}\;. \end{array}$$ (17)

Note that for an intensity of $\hat{p}=1$ over a time *T* = *t*_*c*_ one has *v* = *v*_*m*_, i.e., the velocity *v*_*m*_ corresponds to the crossover power, as expected. When instead of time the distance of the run is fixed, a similar expression for the velocity can be derived. Setting *T* = *d*/*v* in Eq (16) and solving for *v*, one finds

$$\begin{array}{c} v\left(\hat{p},d\right)=\{\begin{array}{cc} -\hat{p}\;v_{m}\gamma_{l}W_{-1}[-\frac{1}{\hat{p}\;\gamma_{l}}\frac{d}{d_{c}}e^{-1/\gamma_{l}}] & \;\text{for}\;\;d\geq d_{c}\\ -\hat{p}\;v_{m}\gamma_{s}W_{-1}[-\frac{1}{\hat{p}\;\gamma_{s}}\frac{d}{d_{c}}e^{-1/\gamma_{s}}] & \;\text{for}\;\;d\leq d_{c} \end{array}\;, \end{array}$$ (18)

where again *d*_*c*_ = *v*_*m*_*t*_*c*_. For the intensity $\hat{p}=1$ this result corresponds to the race velocity of Eq (12). It is important to stress that running economy and endurance both depend on the absolute values for basal and crossover power, *P*_*b*_ and *P*_*m*_, but race times and paces are determined only by the physiological parameters *v*_*m*_, *γ*_*l*_, *γ*_*s*_ and *t*_*c*_. In Application 2 below we demonstrate the dependence of race paces on the physiological parameters of an athlete.

## Results

### Physiological model parameters from records

Previously, accurate models for running performance have been based on a combination of empirical data descriptions and underlying physiological processes, or they employed at least some empirical correction factors. Data like world record performances contain very useful information about maximized physiological response, and can be used to validate theoretical models that have been derived entirely from bio-energetic considerations. Our model fulfills this requirement, and in this section we shall validate its accuracy by comparing it to various record performances.

World and other records have been analyzed before and found to follow an approximate power law. However, the exponent of this power law shows variations with gender and distance which renders its universality and general applicability questionable. Also, there is no physiological foundation for a simple power law. In fact, the existence of a crossover velocity *v*_*m*_ implies different scaling of performances below and above this velocity due to distinct physiological and bio-energetic processes involved.

We have analyzed record performances for eight distances, from 1000m to the marathon, for world records (current as of Oct. 2018, 2000, 1990, and 1980), current European records, and current national records (USA, Germany) see Table 1 for male records, and Table 2 for female records. Following the method described in the previous section, we have estimated the parameters of our model for each group of records. The resulting parameters *t*_*c*_, *v*_*m*_, *γ*_*s*_, and *γ*_*l*_ together with the endurances *E*_*s*_ and *E*_*l*_ are summarized in Tables 1 and 2. The mean relative error between our model prediction and the VDOT prediction for the race times for 13 distances between 1000m and the marathon are 0.15%, 0.11%, and 0.18% for VDOT = 40, 60, and 80, respectively. These small errors suggest that the race times predicted by the VDOT model are mutually consistent. This presumably reflects that the times were obtain from a mathematical model that is based on physiological observations made by Daniels among well trained and elite runners.

**Table 1:** Race times and model parameters for various male running records, as of Oct. 2018.

Record	WR men			WR 2000 men			WR 1990 men		
*t*_*c*_[min]		6.26			5.50			5.90	
*v*_*m*_[m/min]		411.72			417.07			405.00	
100 *γ*_*s*_		9.99			9.87			11.76	
100 *γ*_*l*_		5.36			6.19			5.93	
*E*_s_		0.37			0.36			0.43	
*E*_*l*_		6.46			5.04			5.41	
distance	*T*	*T*_model_	%	*T*	*T*_model_	%	*T*	*T*_model_	%
1000	02:11.96	02:11.94	-0.02	02:11.96	02:11.94	-0.02	02:12.80	02:12.82	+0.01
1500	03:26.00	03:26.24	+0.12	03:26.00	03:26.24	+0.12	03:29.46	03:29.26	-0.10
1609.34	03:43.13	03:42.91	-0.10	03:43.13	03:42.91	-0.10	03:46.32	03:46.50	+0.08
3000	07:20.67	07:20.99	+0.07	07:20.67	07:19.38	-0.29	07:29.45	07:30.88	+0.32
5000	12:37.35	12:37.10	-0.03	12:39.36	12:38.37	-0.13	12:58.39	12:56.88	-0.19
10000	26:17.53	26:18.84	+0.08	26:22.75	26:33.98	+0.71	27:08.23	27:08.68	+0.03
21097.5	58:23.00	58:11.94	-0.32	59:22.00	59:18.82	-0.09	1:00:46.00	1:00:25.03	-0.58
42195	2:01:39.00	2:01:52.99	+0.19	2:05:42.00	2:05:26.57	-0.20	2:06:50.00	2:07:21.71	+0.42
mean			0.12			0.21			0.22
Record	WR 1980 men			US men			EU men		
*t*_*c*_[min]		5.26			6.08			4.97	
*v*_*m*_[m/min]		405.27			406.06			412.81	
100 *γ*_*s*_		12.74			10.35			12.24	
100 *γ*_*l*_		6.21			5.67			5.76	
*E*_s_		0.46			0.38			0.44	
*E*_*l*_		5.00			5.83			5.67	
distance	*T*	*T*_model_	%	*T*	*T*_model_	%	*T*	*T*_model_	%
1000	02:13.40	02:13.41	+0.01	02:13.90	02:13.87	-0.02	02:12.18	02:12.17	-0.01
1500	03:31.36	03:31.26	-0.05	03:29.30	03:29.61	+0.15	03:28.81	03:28.90	+0.04
1609.34	03:48.80	03:48.89	+0.04	03:46.91	03:46.62	-0.13	03:46.32	03:46.24	-0.04
3000	07:32.10	07:34.44	+0.52	07:29.00	07:28.52	-0.11	07:26.62	07:26.39	-0.05
5000	13:08.40	13:04.65	-0.48	12:53.60	12:51.55	-0.26	12:49.71	12:48.61	-0.14
10000	27:22.47	27:30.09	+0.46	26:44.36	26:53.60	+0.58	26:46.57	26:49.72	+0.20
21097.5	1:02:16.00	1:01:26.52	-1.32	59:43.00	59:41.08	-0.05	59:32.00	59:38.51	+0.18
42195	2:09:01.00	2:10:02.03	+0.79	2:05:38.00	2:05:26.28	-0.16	2:05:48.00	2:05:34.12	-0.18
mean			0.46			0.18			0.11
Record	GER men								
*t*_*c*_[min]		4.79							
*v*_*m*_[m/min]		411.05							
100 *γ*_*s*_		11.22							
100 *γ*_*l*_		6.11							
*E*_s_		0.41							
*E*_*l*_		5.14							
distance	*T*	*T*_model_	%						
1000	02:14.53	02:14.52	-0.01						
1500	03:31.58	03:31.71	+0.06						
1609.34	03:49.22	03:49.10	-0.05						
3000	07:30.50	07:30.28	-0.05						
5000	12:54.70	12:57.09	+0.31						
10000	27:21.53	27:13.07	-0.52						
21097.5	1:00:34.00	1:00:45.41	+0.31						
42195	2:08:33.00	2:08:28.19	-0.06						
mean			0.17						

**Table 2:** Race times and model parameters for various female records, as of Oct. 2018. ^†^ For the women WR of 2000 the result of Chinese runners for the distances 1500m, 3000m, 5000m and 10000m have been excluded due to use of performance-enhancing drugs [30].

Record	WR women			WR 2000 women^†^			WR 1990 women		
*t*_*c*_[min]		8.30			10.01			5.50	
*v*_*m*_[m/min]		361.37			352.14			364.74	
100 *γ*_*s*_		9.60			10.27			12.13	
100 *γ*_*l*_		4.85			5.53			5.74	
*E*_s_		0.35			0.38			0.44	
*E*_*l*_		7.88			6.10			5.70	
distance	*T*	*T*_model_	%	*T*	*T*_model_	%	*T*	*T*_model_	%
1000	02:28.98	02:28.78	-0.13	02:28.98	02:29.07	+0.06	02:30.67	02:30.17	-0.33
1500	03:50.07	03:52.05	+0.86	03:52.47	03:52.94	+0.20	03:52.47	03:57.27	+2.06
1609.34	04:12.56	04:10.70	-0.74	04:12.56	04:11.75	-0.32	04:21.68	04:16.95	-1.81
3000	08:20.68	08:18.13	-0.51	08:21.64	08:21.94	+0.06	08:22.62	08:25.93	+0.66
5000	14:11.15	14:12.40	+0.15	14:31.48	14:29.77	-0.20	14:37.33	14:31.08	-0.71
10000	29:17.45	29:29.07	+0.66	30:13.74	30:14.88	+0.06	30:13.74	30:24.18	+0.58
21097.5	1:04:51.00	1:04:50.60	-0.01	1:06:40.00	1:06:56.85	+0.42	1:08:32.00	1:07:34.87	-1.39
42195	2:15:25.00	2:15:00.95	-0.30	2:20:43.00	2:20:18.48	-0.29	2:21:06.00	2:22:16.17	+0.83
mean			0.42			0.20			1.05
Record	US women			EU women			GER women		
*t*_*c*_[min]		10.80			10.19			5.87	
*v*_*m*_[m/min]		347.42			351.63			356.56	
100 *γ*_*s*_		9.39			10.25			13.91	
100 *γ*_*l*_		5.17			4.63			5.01	
*E*_s_		0.34			0.38			0.49	
*E*_*l*_		6.92			8.66			7.35	
distance	*T*	*T*_model_	%	*T*	*T*_model_	%	*T*	*T*_model_	%
1000	02:31.80	02:32.01	+0.14	02:28.98	02:29.08	+0.07	02:30.67	02:30.48	-0.13
1500	03:56.29	03:56.68	+0.17	03:52.47	03:52.92	+0.20	03:57.71	03:59.58	+0.79
1609.34	04:16.71	04:15.62	-0.42	04:12.56	04:11.73	-0.33	04:21.59	04:19.83	-0.67
3000	08:25.83	08:26.40	+0.11	08:21.42	08:21.75	+0.07	08:29.89	08:34.62	+0.93
5000	14:38.92	14:37.27	-0.19	14:23.75	14:27.22	+0.40	14:42.03	14:41.99	-0.00
10000	30:13.17	30:24.74	+0.64	29:56.34	29:56.03	-0.02	30:57.00	30:34.60	-1.21
21097.5	1:07:34.00	1:07:03.57	-0.75	1:06:25.00	1:05:40.14	-1.13	1:07:58.00	1:07:25.42	-0.80
42195	2:19:36.00	2:20:00.22	+0.29	2:15:25.00	2:16:23.60	+0.72	2:19:19.00	2:20:46.06	+1.04
mean			0.34			0.37			0.70

A number of interesting observations can be made from the results: There is a high level of agreement between actual and predicted times with the relative error being larger than 1% only for a single event (Half-marathon, WR 1980) for male records, and four events for female records. The mean of the absolute value of the relative error is always smaller than 1% with the exception of the female WR from 1990 where it is 1.05%. For the male WR a decrease of the absolute value of the relative error from 1980 to today can be observed, indicating an increasing optimization towards the maximally possible performance (within current level of technology and training methods) that is described by our model. Hence, the record times have become more consistent with our model over time which might be also due to an increasing number of attempts to achieve best possible performances. A similar observation is made for the female WR from 1990 to 2000. However, from the 2000 WR some results (Chinese runner’s results for 1500m, 3.000m, 5.000m, and 10.000m) have been excluded due to the use of performance-enhancing drugs [30], and the current WR for 1500m and 10.000m are also controversial [31]. For the latter two distances our model predicts more than 0.5% slower times than actually raced. It is interesting to observe that our predictions are very sensitive to exceptional performances for a particular distance compared to the other distances, and hence is able to identify suspicious race results. Due to the women’s shorter history of endurance running, the female world records for 1980 are less consistent than more recent records and hence have been excluded them from our analysis.

It also instructive to compare the physiological model parameters obtained from the record performances. For the male records, the obtained values for *t*_*c*_ vary between five and six minutes, which is in very good agreement with laboratory testing [32]. However, for female records, we observe a larger variation in *t*_*c*_ with values around 10min being not unusual. However, in cases with such long *t*_*c*_ the crossover velocity *v*_*m*_ is reduced proportionally. The endurance parameter *E*_*l*_ for long distances varies between 5 and 6 for male records, implying that 90% of maximal aerobic power can be maintained for a duration between approximately 25min and 36min, for the values of *t*_*c*_ observed here. For female records, the endurance parameter *E*_*l*_ is significantly larger with variations in an interval of approximately 6 to 8.5, implying that 90% of maximal aerobic power can be maintained for durations up to 85min.

**Fig 2:**
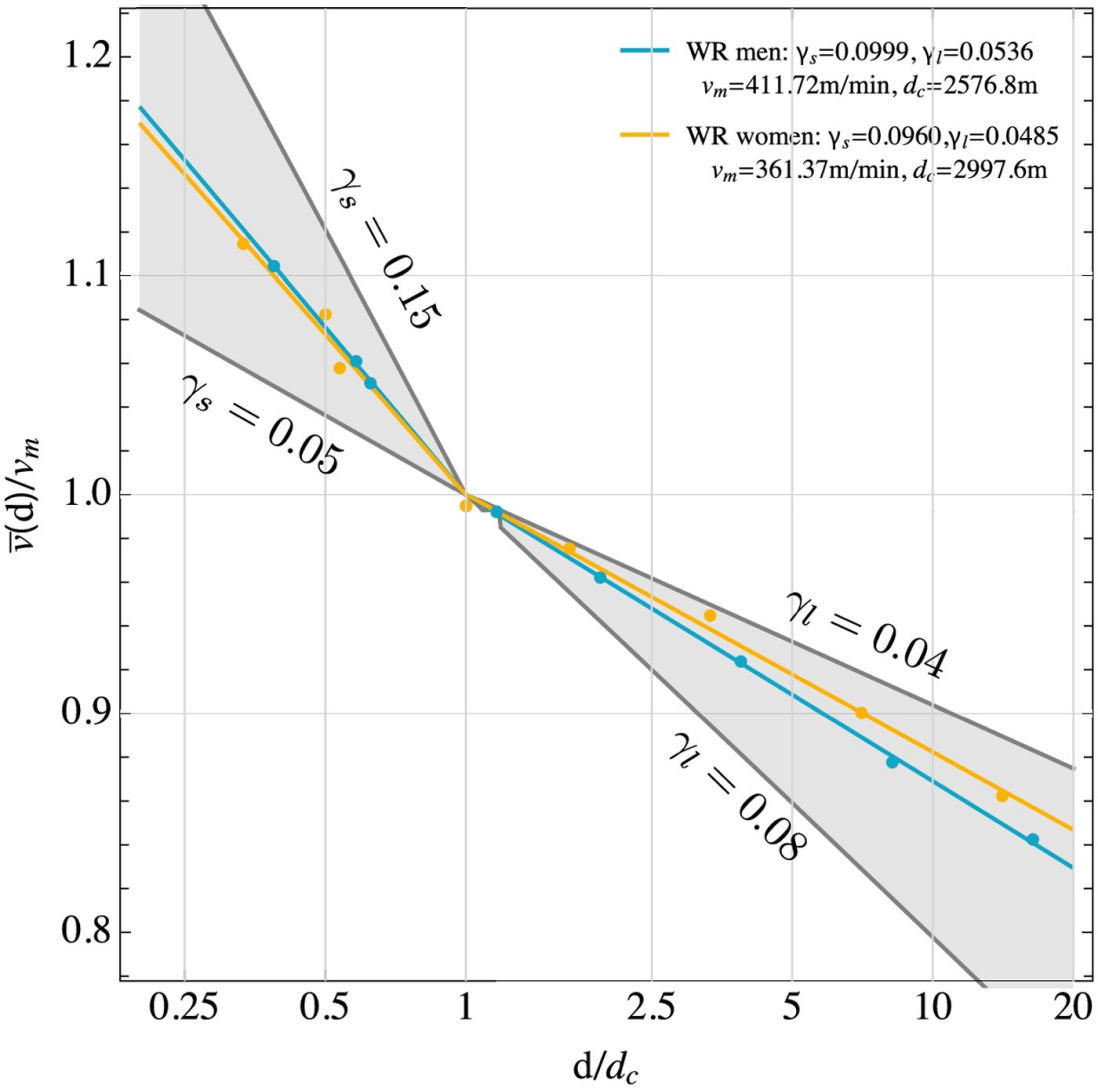
Mean race velocity $\bar{v}\left(d\right)$ as function of race distance. Velocity is re-scaled by *v*_*m*_, and distance *d* is re-scaled by *d*_*c*_ = *v*_*m*_*t*_*c*_. Shown are the male and female world records (WR, dots), model prediction from Eq (12) (solid lines), and a typically expected maximal range of velocities (gray regions). Indicated are the lower and upper limits of *γ*_*s*_ and *γ*_*l*_ for these regions. Due to the re-scaling of $\bar{v}\left(d\right)$ and *d*, this graph highlights endurance for short and long duration, independently of the velocity *v*_*m*_ at maximal aerobic power.

The impact of endurance alone on running performances can be highlighted by measuring the mean race velocity $\bar{v}\left(d\right)$ in units of the crossover velocity *v*_*m*_ and the race distance *d* in units of the crossover distance *d*_*c*_ = *v*_*m*_*t*_*c*_. The resulting relation between $\bar{v}\left(d\right)/v_{m}$ and *d*/*d*_*c*_ is shown in Fig 2 for the current world records. Our model predicts that this relation depends only on the endurance parameters *γ*_*l*_ and *γ*_*s*_, see Eq (12). The corresponding model curves are also plotted in Fig 2, showing good agreement with the data from world records. The better endurance of women for distances longer than the crossover distance *d*_*c*_ is clearly visible. The gray cones in the figure indicate the range of endurance parameters that could potentially be realized in practice by runners from the recreational to the elite level with suitable event specialization. This type of visualization of race performances allows one to evaluate runner’s endurance independently of their maximal aerobic power and running economy which are described by the parameters *v*_*m*_ and *t*_*c*_.

### Estimate of supplemental power

**Fig 3:**
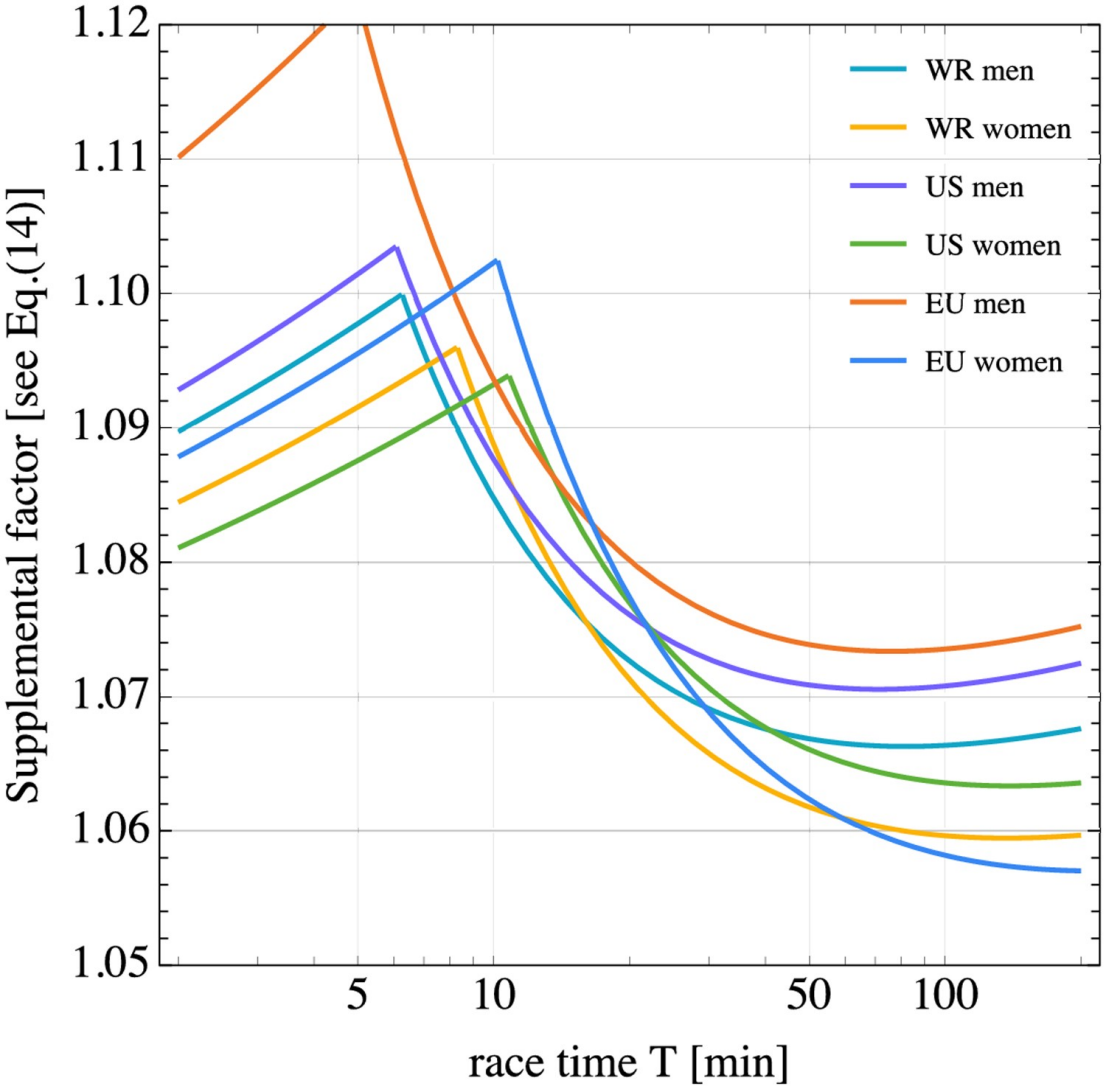
Plot of the supplemental factor of Eq (15) for as predicted by our model for male and female world records (WR), US records (US), and European records (EU). The cusp in the curves occurs at the time *t*_*c*_.

We have seen that supplemental power is responsible for a slow logarithmic decline of racing velocities with distance. In Fig 3 the supplemental factor of Eq (15) (square brackets in this equation) is plotted for various record performances as function of the race duration *T*. The variation range of the factor implies a supplemental power between ≈ 6% and 10% above the nominal power, with the European male records (EU men) being an outlier. The curves have their maximum at the crossover time *T* = *t*_*c*_. During supra-maximal exercise (for times shorter than *t*_*c*_), the oxygen uptake cannot stabilize and continues to increase until the end of the race [33]. Hence we observe an increasing deviation from the nominal power with increasing duration. However, at very short times below about 1 minute, oxygen uptake kinetics limit oxygen supply, and the energy deficit is compensated by the anaerobic system. After 30 to 60 seconds, the oxygen uptake can reach 90% of VO_2*max*_ [33]. This short term kinetic effect is not included in our model. Above *t*_*c*_, i.e., for sub-maximal velocities, oxygen uptake stabilizes and the supplemental factor decreases. However, it does not decrease to one and this is likely related to the fact that the energy cost of running starts to increase above a nominal linear curve when the lactate threshold is approached [34]. For even longer race durations, we observe a slight increase in the supplemental factor that is presumably linked to the increase of the energy cost of running with increasing distance, as discussed in the Introduction. For a marathon or a 2 hour run at about 80% VO_2*max*_ the supplemental power was measured to be between 5% and 7% in terms of oxygen uptake [35, 36] which is consistent with our model prediction for *T* ∼ 120min. We note that for male records, the supplemental factor shows a shallow minimum around one hour. For female records this minimum is displaced to times above two hours.

### Application 1: Reconstruction individual physiological profiles

After we have validated the accuracy of our model against record performances, we would like to find out if it can be also applied to individual runners. If that is the case then one could compute from their personal best performances their individual physiological parameters that characterize their training state and future performance potential. The assessment of the training state of an individual is important not only for performance optimization but also beyond competitive athletics for the monitoring of the health status of recreational runners.

There have been performance models developed for individual runners. A popular model is the so-called VDOT model by Daniels [37]. This model and other approaches employ maximal oxygen uptake as single factor determining performance [38, 39] and this parameter is then used to determine the training state and to predict running performances. A notable exception is the model Peronnet and Thibault which has been also applied to individual runners [7]. It turns out that their model yields comparable but somewhat larger errors than the present model. Partially, this might be due their model’s assumption that the energy cost of running and the crossover time *t*_*c*_ would be identical for all runners. Other physiological factors that determine an individual’s performance include blood lactate concentration, and the anaerobic threshold. However, these parameters require laboratory measurements that are not always available, particularly on sufficiently short time intervals and for recreational athletes.

With the advent of large online databases for personal best performances, it becomes possible to probe the accuracy of performance models for a large set of individual athletes. Similar to our analysis of running records, our model predictions for individual runners can be validated through comparison with their personal best performances. First we reconstruct running economy and endurance profiles of an individual runner from personal best performances for a few race distances and then estimate projected race times for other distances and also some characteristic paces. This eliminates physiological uncertainties that result from the use of universal, typical physiological parameters in previous models. In fact, the present model provides a general scheme that can be applied to any endurance runner over a range of distances and it is not based on observations made for only a small sample of trained athletes. Our approach also yields individual relative intensities, in percent of the aerobic power reserve *P*_*m*_ − *P*_*b*_, at which a runner performs races. This is important for the relative use of fat and carbohydrate as fuels, and hence the total carbohydrate consumption for a given race distance.

In the following, we apply our model to personal best performances of British runners that are available online in the database www.thepowerof10.info [40]. As a first test of our model for individual runners, we have considered the personal bests of the top nine male and female marathon runners from this database, according to the 2015 ranking. Their personal best times for seven distances from 800m to the marathon are summarized in Tables 3 and 4. With the same methodology that we used for running records above, we obtain the four model parameters for each runner that are also listed in the tables. From these parameters we compute the predicted race times. We find that the agreement between the predicted and actual race times are the most accurate to date, with an average mean error of less than 1% for each individual runner for all seven distances, see Tables 3 and 4. This suggests that our model can describe the running performance of individual runners with reliable accuracy. The slightly larger mean error for individuals than for groups of runners (record holders) appears natural since an individual runner can hardly reach optimized performance for all distances. When analyzing personal bests of an individual runner one should also realize that the best times on various distances have been probably obtained over a large time span of many years. Especially at the beginning of the career of a runner, when he races predominantly shorter distances, performance might not be optimal. Alternatively, one could consider only best performances obtained within a short time interval like a year which however limits presumably the available distances.

**Table 3:** Personal best times and model parameters for individuals (Leading male marathon runners from UK, ranking 2015, http://www.thepowerof10.info/rankings).

Runner		01			02			03	
*t*_*c*_[min]		23.84			11.28			4.57	
*v*_*m*_[m/min]		353.28			360.35			373.49	
100 *γ*_*s*_		8.16			11.65			10.26	
100 *γ*_*l*_		4.67			5.07			4.85	
*E*_s_		0.29			0.42			0.38	
*E*_*l*_		8.52			7.20			7.86	
distance	*T*	*T*_model_	%	*T*	*T*_model_	%	*T*	*T*_model_	%
800	01:52.08	01:52.52	+0.39	01:49.98	01:49.94	-0.04	01:58.32	01:58.32	+0.00
1500	03:41.88	03:41.06	-0.37	03:40.80	03:40.95	+0.07	03:57.48	03:57.48	-0.00
3000	07:48.90	07:46.84	-0.44	08:00.48	08:00.34	-0.03	08:16.62	08:16.24	-0.08
5000	13:28.32	13:31.65	+0.41	13:57.66	14:01.83	+0.50	14:13.32	14:09.91	-0.40
10000	28:49.02	28:32.80	-0.94	29:23.04	29:09.19	-0.79	29:18.48	29:26.13	+0.43
21097.5	1:01:25.02	1:02:32.11	+1.82	1:04:07.02	1:04:12.25	+0.14	1:04:30.00	1:04:49.91	+0.51
42195	2:10:55.02	2:09:41.73	-0.93	2:13:40.98	2:13:52.45	+0.14	2:15:51.00	2:15:11.79	-0.48
mean			0.76			0.24			0.27
Runner		04			05			06	
*t*_*c*_[min]		19.88			8.57			6.88	
*v*_*m*_[m/min]		357.87			349.94			382.82	
100 *γ*_*s*_		8.27			4.84			6.93	
100 *γ*_*l*_		5.70			4.19			5.70	
*E*_s_		0.30			0.13			0.24	
*E*_*l*_		5.78			10.86			5.79	
distance	*T*	*T*_model_	%	*T*	*T*_model_	%	*T*	*T*_model_	%
800	01:51.78	01:52.20	+0.38	02:09.48	02:08.54	-0.73	01:55.20	01:55.20	+0.00
1500	03:41.94	03:40.71	-0.56	04:05.22	04:08.44	+1.31	03:45.66	03:45.66	-0.00
3000	07:46.74	07:46.78	+0.01	08:40.50	08:34.37	-1.18	08:00.12	07:53.93	-1.29
5000	13:31.20	13:32.52	+0.16	14:38.58	14:36.90	-0.19	13:33.00	13:35.29	+0.28
10000	28:42.18	28:31.86	-0.60	30:04.02	30:10.06	+0.33	27:57.24	28:25.13	+1.66
21097.5	1:02:22.98	1:03:06.46	+1.16	1:04:46.98	1:05:55.65	+1.77	1:03:00.00	1:03:04.39	+0.12
42195	2:12:57.00	2:12:10.52	-0.58	2:18:21.00	2:16:24.13	-1.41	2:13:40.02	2:12:33.95	-0.82
mean			0.49			0.99			0.60
Runner		07			08			09	
*t*_*c*_[min]		8.44			8.17			5.28	
*v*_*m*_[m/min]		355.63			367.03			347.39	
100 *γ*_*s*_		5.72			8.15			15.25	
100 *γ*_*l*_		5.62			5.81			4.82	
*E*_s_		0.17			0.29			0.52	
*E*_*l*_		5.93			5.59			7.95	
distance	*T*	*T*_model_	%	*T*	*T*_model_	%	*T*	*T*_model_	%
800	02:05.10	02:04.97	-0.11	01:57.42	01:57.12	-0.26	02:00.42	02:00.42	+0.00
1500	04:02.40	04:02.87	+0.19	03:49.98	03:51.04	+0.46	04:10.08	04:10.08	-0.00
3000	08:28.62	08:26.15	-0.49	08:14.04	08:10.42	-0.73	08:47.70	08:51.43	+0.71
5000	14:35.94	14:30.06	-0.67	14:01.02	14:04.01	+0.36	15:18.30	15:09.93	-0.91
10000	30:04.02	30:17.72	+0.76	29:32.70	29:26.30	-0.36	31:30.90	31:30.08	-0.04
21097.5	1:06:04.02	1:07:09.06	+1.64	1:04:28.02	1:05:23.05	+1.42	1:09:12.00	1:09:20.92	+0.21
42195	2:22:55.98	2:20:56.89	-1.39	2:18:49.02	2:17:31.77	-0.93	2:24:31.02	2:24:32.68	+0.02
mean			0.75			0.65			0.27

**Table 4:** Personal best times and model parameters for individuals (Leading female marathon runners from UK, ranking 2015, http://www.thepowerof10.info/rankings).

Runner		01			02			03	
*t*_*c*_[min]		13.01			9.48			3.85	
*v*_*m*_[m/min]		319.66			316.60			368.12	
100 *γ*_*s*_		5.45			6.80			5.82	
100 *γ*_*l*_		4.70			4.28			4.41	
*E*_s_		0.16			0.23			0.18	
*E*_*l*_		8.39			10.34			9.64	
distance	*T*	*T*_model_	%	*T*	*T*_model_	%	*T*	*T*_model_	%
800	02:17.28	02:17.17	-0.08	02:18.60	02:18.31	-0.21	02:05.94	02:05.94	-0.00
1500	04:25.56	04:25.95	+0.15	04:29.58	04:30.60	+0.38	04:05.40	04:05.12	-0.11
3000	09:13.08	09:12.71	-0.07	09:32.82	09:28.54	-0.75	08:22.20	08:26.50	+0.86
5000	15:44.22	15:47.11	+0.31	16:13.02	16:09.74	-0.34	14:29.10	14:25.36	-0.43
10000	32:39.36	32:42.02	+0.14	33:01.98	33:23.16	+1.07	30:01.08	29:51.82	-0.51
21097.5	1:12:36.00	1:11:45.67	-1.16	1:12:28.02	1:13:01.34	+0.77	1:05:40.02	1:05:30.03	-0.25
42195	2:28:04.02	2:29:05.72	+0.69	2:32:40.02	2:31:12.50	-0.96	2:15:25.02	2:16:00.78	+0.44
mean			0.37			0.64			0.37
Runner		04			05			06	
*t*_*c*_[min]		9.45			5.92			12.36	
*v*_*m*_[m/min]		317.48			297.47			303.80	
100 *γ*_*s*_		6.93			9.70			9.00	
100 *γ*_*l*_		5.06			4.62			6.19	
*E*_s_		0.24			0.36			0.33	
*E*_*l*_		7.23			8.71			5.02	
distance	*T*	*T*_model_	%	*T*	*T*_model_	%	*T*	*T*_model_	%
800	02:18.72	02:17.68	-0.75	02:28.80	02:28.80	-0.00	02:17.40	02:17.16	-0.18
1500	04:26.04	04:29.59	+1.33	04:57.42	04:57.42	-0.00	04:30.84	04:31.69	+0.31
3000	09:36.72	09:26.96	-1.69	10:22.86	10:21.12	-0.28	09:40.44	09:39.64	-0.14
5000	16:08.10	16:11.38	+0.34	17:43.02	17:42.23	-0.07	16:47.82	16:46.53	-0.13
10000	33:24.72	33:39.59	+0.74	36:40.02	36:42.61	+0.12	35:18.00	35:11.88	-0.29
21097.5	1:13:21.00	1:14:10.86	+1.13	1:19:55.02	1:20:39.07	+0.92	1:17:43.02	1:18:25.22	+0.90
42195	2:36:39.00	2:34:47.30	-1.19	2:48:55.98	2:47:45.34	-0.70	2:46:19.02	2:45:29.11	-0.50
mean			1.03			0.30			0.35
Runner		07			08			09	
*t*_*c*_[min]		16.50			5.40			14.64	
*v*_*m*_[m/min]		281.73			300.16			272.55	
100 *γ*_*s*_		7.43			16.76			7.25	
100 *γ*_*l*_		4.28			5.16			4.45	
*E*_s_		0.26			0.55			0.25	
*E*_*l*_		10.33			6.93			9.47	
distance	*T*	*T*_model_	%	*T*	*T*_model_	%	*T*	*T*_model_	%
800	02:29.82	02:29.38	-0.29	02:20.22	02:20.22	-0.00	02:37.26	02:36.54	-0.46
1500	04:51.42	04:52.95	+0.52	04:55.20	04:55.20	-0.00	05:04.32	05:06.82	+0.82
3000	10:18.72	10:17.25	-0.24	10:08.70	10:20.47	+1.93	10:48.48	10:46.06	-0.37
5000	17:58.98	17:48.35	-0.98	18:13.98	17:44.85	-2.66	18:26.70	18:32.44	+0.52
10000	36:31.98	36:45.40	+0.61	37:07.98	36:59.37	-0.39	38:34.98	38:19.98	-0.65
21097.5	1:19:07.02	1:20:19.99	+1.54	1:20:39.00	1:21:45.39	+1.37	1:24:06.00	1:23:55.81	-0.20
42195	2:48:16.02	2:46:13.19	-1.22	2:51:46.02	2:51:06.21	-0.39	2:53:25.02	2:53:58.66	+0.32
mean			0.77			0.96			0.48

**Fig 4:**
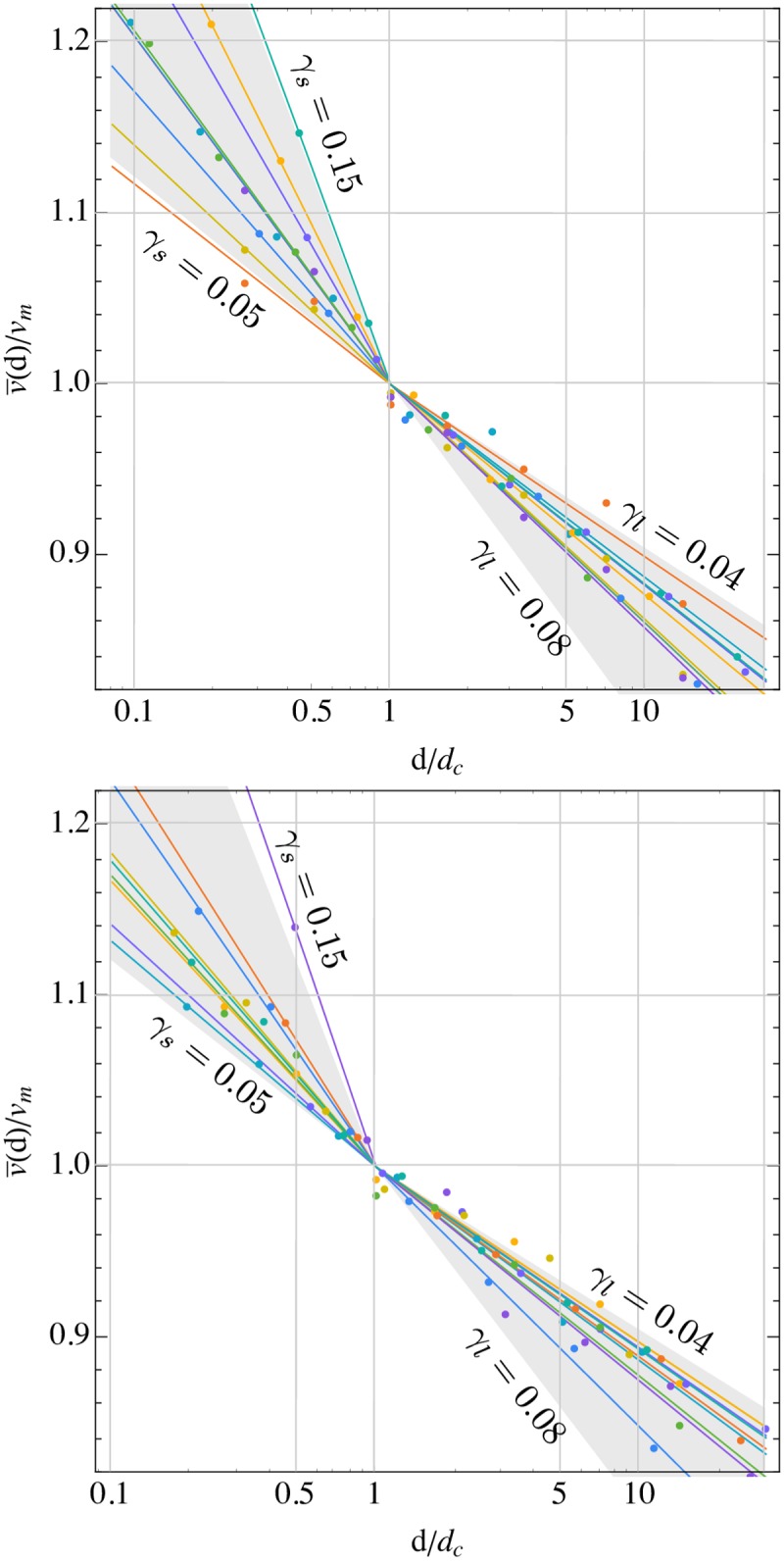
Same visualization of endurance as in Fig 2 but for individual male (top) and female (bottom) runners, see Tables 3 and 4. Colors label different runners.

Hence the individual variations of the parameters *t*_*c*_ and *v*_*m*_ can be large but they are strongly correlated. This suggests that *t*_*c*_ gives a rather precise estimate of the time over which a runner can sustain the velocity *v*_*m*_ which, however, can deviate slightly from the actual velocity at VO_2*max*_, depending on the available personal best performances in the vicinity of this crossover point. In order to measure individual endurances independently of aerobic capacity, we have computed and plotted the relation between the re-scaled race velocity $\bar{v}\left(d\right)/v_{m}$ and distance *d*/*d*_*c*_ in analogy to our analysis of running records, see Fig 4. Two important observations can be made from this graph: (1) For each individual runner, there are two distinct relations between velocity and distance above and below the crossover velocity *v*_*m*_ and distance *d*_*c*_. (2) Even within the group of top UK marathon runners, there is a large variation in endurances as quantified by the different slopes of the re-scaled velocity-distance curves and the parameters *γ*_*s*_ and *γ*_*l*_. They gray cones of expected maximal variations shown in Fig 4 are almost completely covered by the performances of the studied runners.

For one of the female runners included in Table 4, runner 03 which is Paula Radcliffe, physiological data are available for a long time span of about 12 years [41]. While her personal records have been obtained over a similar period of time (800m in 1993 and marathon in 2003), and her physiological data have progressed during this time, in particular running economy, we can compare our model prediction for the speed *v*_*m*_ to Radcliffe’s speed at VO2max, averaged over the time period from 1993 to 2003 which is about 22.5 km/h or 375.0 m/min [41]. This value compares very well with our finding of *v*_*m*_ = 373.5 m/min, see Table 4.

Our findings show that individual performances do not follow a unique power law as suggested, for example, by Riegel’s formula. There are more complex variations of physiological metrics among runners and those have to be taken into account for describing and predicting accurately performances and presumably optimal training. Our computational approach reveals the physiological parameters that determine individual performance and explains how they can be used in praxis to guide training and racing.

### Application 2: Personalized characteristic paces

We expect that our four parameter model can measure an individual runner’s performance status for distances from 800m to the marathon more accurately than previous performance models that often assume for all runners the same (average) values for certain characteristics like running economy or endurance. An example for the latter type of models is the popular VDOT model of J. Daniels which assumes a fixed running economy and endurance curves for all runners [37, 42]. Although the VDOT model represents a good first approximation of characteristic paces based on a single race performance, the ability to monitor individual performances with more than just one parameter allows the runner to ascertain a better understanding of their training status and potential performance. It then becomes beneficial to have a model that makes use of larger available data sets. In the same way that one may better understand current fitness by examining relative oxygen consumption at different paces rather than absolute oxygen consumption, [43] developing an approach that makes use of performance over several races describes an individual runner better than a single race.

Characteristic paces are often defined by the pace that a runner can race (at current training status) for a prescribed duration or distance. When the physiological model parameters of a runner are known from sufficiently many recent race performances, the running velocities for a prescribed intensity and duration, or intensity and distance can be computed from Eqs (17) and (18), respectively. In the following we consider race paces for a given duration or distance, corresponding to $\hat{p}=1$ in these equations. In order to compare our model predictions to the characteristic paces of the VDOT model, we consider three hypothetical runners that are assumed to have achieved race performances as predicted by the VDOT model with model parameter values VDOT = 40, 60, and 80. (VDOT can be regarded as an effective value for VO_2*max*_, see [37] for details.) From these race performances we obtain the four parameters of our model. These parameters are given in the captions of Tables 5, 6 and 7. These tables provide race paces (time per km) for various distances and durations specified in the first column. Some of the paces correspond the specific paces named in the VDOT model, and they are labeled correspondingly as R-, I-, T- and M-pace. The paces proposed by the VDOT model are given in the second column. The remaining columns provide the predictions of our model. The third column lists the paces as obtained from the values of the four model parameters that result from the hypothetical race performances of the runner with the given VDOT score. There is agreement within a few seconds per kilometer. It should be kept in mind that our model, unlike the VDOT model, does not implement any fixed parameters or constants a priori. We observe that the fixed parameters of the VDOT model correspond to rather superior endurance with *γ*_*l*_ ≈ 0.05 for long distances and average endurance with *γ*_*s*_ ≈ 0.09 for short distances. As we have seen above, there is substantial variation in these parameters among individuals. Hence, characteristic paces should also determined individually. We have modified the endurance parameters *γ*_*l*_ and *γ*_*s*_ independently within their typical minimal and maximal values while keeping *v*_*m*_ and *t*_*c*_ unchanged. The resulting paces are shown in the last four columns of the tables. The fast paces for short distances (1mile and 5min paces) can change up to ±10sec/km compared to the original VDOT model which is substantial. For the slower paces (for time *t*_*c*_ and longer) the variation can be even larger with a maximum change for the marathon pace (M-pace). For a VDOT = 40 runner, the M-pace window between slowest and fastest pace is about 55sec/km, for a VDOT = 60 runner it is about 30sec/km and even for a high level runner with VDOT = 80 it is still about 20sec/km. These variations result from different endurances, with the crossover speed *v*_*m*_ unchanged. We have also studied the effect of a modification of the time *t*_*c*_ from the original VDOT model value which appears rather long with 12 to 13min. The results are shown in Tables 8, 9 and 10. The first three columns have the same meaning as in the three tables before. The last four columns list the paces that correspond to a reduction or an increase of *t*_*c*_ by 10% or 20%, respectively. Here we observe a smaller variation by a few seconds around the original paces, relatively independent of the duration or distance that defines the pace. This shows that racing paces are more dependent on endurance than on the time over which runners can sustain their crossover speed at VO_2*max*_. The reason for that is the exponential dependence on *γ*_*s*_, *γ*_*l*_ of the duration *T*(*p*) over which a relative power *p* can be maintained, independently of *t*_*c*_ and *v*_*m*_, see Fig 1.

**Table 5:** Paces per km for a runner with VDOT = 40 score for different endurances. The original physiological parameters are *t*_*c*_ = 12.35min, *v*_*m*_ = 214.88m/min, *γ*_*l*_ = 0.051 and *γ*_*s*_ = 0.096. In last 4 columns the endurances *E*_*l*_ and *E*_*s*_ are given only when they are different from the original values.

pace at max. power for	Ref. [37]	original	*γ*_*l*_ = 0.04	*γ*_*l*_ = 0.08	*γ*_*s*_ = 0.15	*γ*_*s*_ = 0.05
		*E*_*l*_ = 7.1	*E*_*l*_ = 12.2	*E*_*l*_ = 3.5		
		*E*_*s*_ = 0.35			*E*_*s*_ = 0.51	*E*_*s*_ = 0.14
1 mile (R-pace)	04:20	04:25.21	orig.	orig.	04:16.72	04:32.06
5min	—	04:16.96	orig.	orig.	04:05.87	04:27.14
time *t*_*c*_ (I-pace)	04:42	04:39.22	orig.	orig.	orig.	orig.
5.000m	04:49	04:49.06	04:46.79	04:55.54	orig.	orig.
10.000m	05:00	05:00.67	04:55.58	05:15.85	orig.	orig.
60min (T-pace)	05:06	05:03.67	04:58.07	05:19.64	orig.	orig.
Half marathon	05:15	05:14.30	05:05.68	05:41.31	orig.	orig.
marathon (M-pace)	05:29	05:28.16	05:15.70	06:09.16	orig.	orig.

**Table 6:** Paces per km for a runner with VDOT = 60 score for different endurances. The original physiological parameters are *t*_*c*_ = 12.67min, *v*_*m*_ = 298.51m/min, *γ*_*l*_ = 0.052 and *γ*_*s*_ = 0.092. The meaning of the columns is the same as in Table 5.

pace at max. power for	Ref. [37]	original	*γ*_*l*_ = 0.04	*γ*_*l*_ = 0.08	*γ*_*s*_ = 0.15	*γ*_*s*_ = 0.05
		*E*_*l*_ = 6.8	*E*_*l*_ = 12.2	*E*_*l*_ = 3.5		
		*E*_*s*_ = 0.34			*E*_*s*_ = 0.51	*E*_*s*_ = 0.14
1 mile (R-pace)	03:05	03:05.04	orig.	orig.	02:54.93	03:12.36
5min	—	03:05.15	orig.	orig.	02:56.39	03:12.07
time *t*_*c*_ (I-pace)	03:23	03:21.00	orig.	orig.	orig.	orig.
5.000m	03:25	03:24.14	03:23.36	03:26.00	orig.	orig.
10.000m	03:32	03:32.41	03:29.49	03:39.64	orig.	orig.
60min (T-pace)	03:40	03:38.78	03:34.33	03:49.55	orig.	orig.
Half marathon	03:42	03:42.12	03:36.53	03:56.62	orig.	orig.
marathon (M-pace)	03:52	03:51.99	03:43.51	04:15.08	orig.	orig.

**Table 7:** Paces per km for a runner with VDOT = 80 score for different endurances. The original physiological parameters are *t*_*c*_ = 12.92min, *v*_*m*_ = 376.85m/min, *γ*_*l*_ = 0.053 and *γ*_*s*_ = 0.088. The meaning of the columns is the same as in Table 5.

pace at max. power for	Ref. [37]	original	*γ*_*l*_ = 0.04	*γ*_*l*_ = 0.08	*γ*_*s*_ = 0.15	*γ*_*s*_ = 0.05
		*E*_*l*_ = 6.6	*E*_*l*_ = 12.2	*E*_*l*_ = 3.5		
		*E*_*s*_ = 0.32			*E*_*s*_ = 0.51	*E*_*s*_ = 0.14
1 mile (R-pace)	02:25	02:23.98	orig.	orig.	02:13.52	02:30.46
5min	—	02:26.99	orig.	orig.	02:19.37	02:32.00
time *t*_*c*_ (I-pace)	02:41	02:39.22	orig.	orig.	orig.	orig.
5.000m	02:40	02:39.45	02:39.39	02:39.59	orig.	orig.
10.000m	02:46	02:45.91	02:44.14	02:49.88	orig.	orig.
60min (T-pace)	02:54	02:53.33	02:49.64	03:01.52	orig.	orig.
Half marathon	02:53	02:53.50	02:49.59	03:02.65	orig.	orig.
marathon (M-pace)	03:01	03:01.22	02:54.99	03:16.46	orig.	orig.

**Table 8:** Paces per km for a runner with VDOT = 40 score for different variations of the time *t*_*c*_. The original physiological parameters are *t*_*c*_ = 12.35min, *v*_*m*_ = 214.88m/min, *γ*_*l*_ = 0.051 and *γ*_*s*_ = 0.096.

pace at max. power for	Ref. [37]	original *t*_*c*_ = 12.35min	0.8*t*_*c*_	0.9*t*_*c*_	1.1*t*_*c*_	1.2*t*_*c*_
1 mile (R-pace)	04:20	04:25.21	04:31.27	04:28.03	04:22.70	04:20.46
5min	—	04:16.96	04:22.12	04:19.37	04:14.82	04:12.90
time *t*_*c*_ (I-pace)	04:42	04:39.22	04:42.43	04:40.73	04:36.70	04:34.43
5.000m	04:49	04:49.06	04:52.69	04:50.76	04:47.53	04:46.15
10.000m	05:00	05:00.67	05:04.62	05:02.52	04:59.02	04:57.52
60min (T-pace)	05:06	05:03.67	05:07.47	05:05.45	05:02.07	05:00.63
Half marathon	05:15	05:14.30	05:18.63	05:16.33	05:12.49	05:10.86
marathon (M-pace)	05:29	05:28.16	05:32.89	05:30.37	05:26.18	05:24.39

**Table 9:** Paces per km for a runner with VDOT = 60 score for different variations of the time *t*_*c*_. The original physiological parameters are *t*_*c*_ = 12.67min, *v*_*m*_ = 298.51m/min, *γ*_*l*_ = 0.052 and *γ*_*s*_ = 0.092.

pace at max. power for	Ref. [37]	original *t*_*c*_ = 12.67min	0.8*t*_*c*_	0.9*t*_*c*_	1.1*t*_*c*_	1.2*t*_*c*_
1 mile (R-pace)	03:05	03:05.04	03:08.94	03:06.86	03:03.42	03:01.97
5min	—	03:05.15	03:08.72	03:06.82	03:03.67	03:02.34
time *t*_*c*_ (I-pace)	03:23	03:21.00	03:23.37	03:22.11	03:19.25	03:17.68
5.000m	03:25	03:24.14	03:26.73	03:25.36	03:23.06	03:22.08
10.000m	03:32	03:32.41	03:35.22	03:33.72	03:31.23	03:30.17
60min (T-pace)	03:40	03:38.78	03:41.60	03:40.10	03:37.60	03:36.54
Half marathon	03:42	03:42.12	03:45.20	03:43.56	03:40.83	03:39.66
marathon (M-pace)	03:52	03:51.99	03:55.36	03:53.57	03:50.58	03:49.31

**Table 10:** Paces per km for a runner with VDOT = 80 score for different variations of the time *t*_*c*_. The original physiological parameters are *t*_*c*_ = 12.92min, *γ*_*l*_ = 0.053 and *γ*_*s*_0.088.

pace at max. power for	Ref. [37]	original *t*_*c*_ = 12.92min	0.8*t*_*c*_	0.9*t*_*c*_	1.1*t*_*c*_	1.2*t*_*c*_
1 mile (R-pace)	02:25	02:23.98	02:26.80	02:25.30	02:22.81	02:21.76
5min	—	02:26.99	02:29.69	02:28.25	02:25.87	02:24.85
time *t*_*c*_ (I-pace)	02:41	02:39.22	02:41.12	02:40.11	02:37.90	02:36.71
5.000m	02:40	02:39.45	02:41.48	02:40.40	02:38.17	02:36.87
10.000m	02:46	02:45.91	02:48.11	02:46.94	02:44.99	02:44.16
60min (T-pace)	02:54	02:53.33	02:55.60	02:54.39	02:52.38	02:51.53
Half marathon	02:53	02:53.50	02:55.91	02:54.63	02:52.49	02:51.58
marathon (M-pace)	03:01	03:01.22	03:03.85	03:02.45	03:00.11	02:59.12

It is interesting to relate this observation to physiological parameters that can be measured in the laboratory and have been linked to endurance capacity, like blood lactate concentration. It is known that the running speed at the lactate threshold can improve independently of VO_2*max*_ and so can the runner’s endurance. Often the lactate threshold pace is identified with the running velocity that a runner can race for about 60min. The corresponding paces are shown in Tables 5–10 as “T-pace”. The relative intensity or power output in percent of the aerobic power reserve [see Eq (1)] at the lactate threshold is given by *p*_*LT*_ = 100[1 − *γ*_*l*_ log(60/*t*_*c*_)]. For example, for a recreational runner (with VDOT = 40), described by the parameters of Table 5, one has *p*_*LT*_ = 91.94% for the original value *γ*_*l*_ = 0.051, while *p*_*LT*_ = 93.68% for *γ*_*l*_ = 0.04, and *p*_*LT*_ = 87.35% for *γ*_*l*_ = 0.08. These values appear rather large when compared to the lactate threshold estimates from current world records: *p*_*LT*_ = 87.08% for male and *p*_*LT*_ = 90.41% for female records. This implies again that the VDOT model assumes a rather optimized endurance.

## Conclusion

Modern performance testing is often based on laboratory testing of athletes with the goal of identifying physiological metrics that correlate with performance and can be linked to fundamental physiological processes. However, measuring physiological metrics requires time consuming and expensive testing, often under rather idealized laboratory conditions. Hence, it appears to be very useful to extract information on power characteristics for individual runners or certain groups of runners from performance results in racing events or time trails. This is of particularly great interest for analyzing the effect of aging on human performance, considering the enormous improvement of performance in older age groups. As stated already by A. V. Hill, world and other records constitute very interesting data sets since their accuracy by far exceeds that of laboratory measurements and they correspond to best human performances at a given time in history under realistic conditions.

The model presented here provides a quantitative method for extracting characteristic parameters from race performances of a group of runners or of an individual runner. The key equations and computational steps of our model are as follows:

- The key equation for the comparison of our model to race results is the expression for the race time *T*(*d*) as function of the race distance *d*, given in Eq 11.
- We minimize the sum of the squared relative deviations in percent between actual race times and the function *T*(*d*) by varying the four model parameters *v*_*m*_, *t*_*c*_, *γ*_*s*_, and *γ*_*l*_ in *T*(*d*) for all distances raced by a runner or a group of runners (records). The final model parameters are those that result from this minimization.
- From the four model parameters the values of which were obtained from race performances or by other input like physiological data, the function *T*(*d*) predicts the race times and Eq 12 the mean race velocities for arbitrary distances.

The model parameters quantify the runner’s performance status and can be used to predict personalized fastest possible but realistic and safe racing paces for a wide range of race distances and durations. Our model provides an unified description of running events at sub- and supra-maximal velocities that are separated by a time scale *t*_*c*_ whose value is in good agreement with independent measurements. On a fundamental level, for the first time our approach provides a derivation of the previously observed but unexplained linear relation between the mean velocity and the logarithm of the duration for running records. The mechanism underlying this logarithmic relation could be identified as the necessity of a supplemental power, beyond the nominal power cost of running, for maintaining the mean velocity. Our findings are different from the previously postulated power law relation between the mean race speed $\bar{v}$ and distance *d*, $\bar{v}∼d^{-\beta }$ with an exponent *β* that varies between 0.054 and 0.083, depending on age and gender [28]. Note that this exponent *β* is slightly smaller than the value 1/8 expected from Kennelly’s original work [1]. A modified, broken power law yielded a crossover duration *t*_*c*_ between 3min and 4min which is too short to be consistent with laboratory measurements [29].

We have validated our model by comparing it to various running records and also to personal records of individual runners. The comparison shows consistently low relative errors between actual and predicted race times, with the mean error being maximally 1% and typically less than that for both world and national records and individual personal records. To our knowledge, this is the to date most accurate theoretical description of running performances that does not require any a priori fixing of physiological constants. The obtain agreement shows that human running performance depends in a subtle manner on several variables that, however, can be quantified for individual runners. Indeed, we find that four parameters can characterize the performance state of a runner: the time *t*_*c*_ over which the velocity *v*_*m*_ can be maintained, and two endurance parameters *E*_*s*_ and *E*_*l*_ for short and long duration endurance. By comparing to independent measurements, we argue that *v*_*m*_ is close to the velocity at maximal aerobic power or VO_2*max*_. By their definition, the endurance parameters yield the duration *E*_*l*_*t*_*c*_ > *t*_*c*_ over which a runner can sustain 90% of *v*_*m*_ or maximal aerobic power and the duration *E*_*s*_*t*_*c*_ < *t*_*c*_ for 110% of *v*_*m*_ or maximal aerobic power.

We have compared our model to Daniel’s VDOT model which is based on a single variable parameter (VDOT) that measures performance. When the race times predicted by the VDOT model are analyzed with our model, we find rather superior long distance endurance parameters *E*_*l*_. For more conservative endurance parameters, our model yields marathon race paces that even for elite runners can be 15sec/km slower than the VDOT predictions. This highlights the importance of proper modeling of individual endurances.

Currently, the possibility of running a sub 2 hour marathon is discussed with great enthusiasm. Our model allows to extract physiological characteristics from race results, like the world records. Using the physiological parameters of the current world records as a basis, we can use our model also to understand to what extend physiological parameters need to progress for a male runner to break 2 hours in the marathon. For example, the latest update of the world record in the marathon by Eliud Kipchoge in Berlin on September 16, 2018 which is included in our results of Table 1, has increased the endurance for long duration from *E*_*l*_ = 5.98 to *E*_*l*_ = 6.46, i.e., by 8%, while the speed that can be raced for 6min (413.5 m/min) and the short term endurance *E*_*s*_ remained basically unchanged. Our model predicts that the endurance for long duration had to be increased to *E*_*l*_ = 7.49 with all other parameters unchanged to obtain a marathon time of 1:59:56. This corresponds to another increase of 16% compared to the just updated value which appears unrealistic in near future. Another possibility, however, would be to assume the endurance of the current world record, *E*_*l*_ = 6.46, and an increased speed at VO2max. For example, increasing the speed that a runner can sustain for 6min by 1.3% to *v*_*m*_ = 418.7 m/min would yield a marathon time of 1:59:58. This could be achieved by an increase in running economy by only ∼1% which seems feasible, at least by material improvements and/or suitable racing conditions (course, climate).

Future studies based on our model could include the dependence of the performance state on distance specialization, altitude, air temperature, age, and other factors. With the availability of big data set on running performances, these studies could be performed with much better statistics than studies with much smaller groups of runners participating in laboratory and clinical studies. Our model could be applied to other endurance sports after a modification of the running specific dependence of power on velocity.

## Supporting information

S1 Appendix Solution of the integral equation for *P*_*max*_(*T*). (PDF)

S2 Appendix Comparison to oxygen uptake measurement. (PDF)
