# Supplementary material for: A minimal power model for human running performance
Source: PLoS One. 2018 Nov 16;13(11):e0206645. doi: 10.1371/journal.pone.0206645 (PMC6239296; doi:10.1371/journal.pone.0206645)
Supplement: S1 Appendix — (PDF) [file pone.0206645.s001.pdf]

S1 Appendix. Solution of the integral equation for $P_{max}(T)$.

The maximal power $P_{max}(T)$ is determined by the integral equation

$$P_{max}(T) + P_{sup}(T) = \frac{1}{T}\int_0^T P_{max}(T-t)dt = \frac{1}{T}\int_0^T P_{max}(t)dt \tag{1}$$

with $P_{sup}(T)$ given by Eq. (3). This equation can be easily transformed into a differential equation by defining the indefinite integral $E(T)$ of $P_{max}(T)$ so that the derivative $E'(T) = P_{max}(T)$. Without loss of generality, we can chose the initial condition $E(0) = 0$. The differential equation for $E(T)$ is then

$$E'(T) + P_{sup}(T) = \frac{E(T)}{T} \tag{2}$$

which has the general solution

$$E(T) = TP_m + TP_{sup}(t_c) - T\int_{t_c}^T \frac{P_{sup}(t)}{t}dt \tag{3}$$

where we imposed the initial condition $E'(T = t_c) = P_m$ so that $P_{max}(T = t_c) = P_m$ as required by definition of P_m. Performing the integral with the constant function $P_{sup}(t) = P_s$ for $T \leq t_c$ yields

$$E(T) = T\left[P_m + P_s - P_s \log(T/t_c)\right] \tag{4}$$

and using $P_{sup}(t) = P_l(t - t_c)/t + P_s t_c/t$ for $T \geq t_c$ yields

$$E(T) = T\left[P_m + P_s + (P_l - P_s)\frac{T - t_c}{T} - P_l \log(T/t_c)\right] . \tag{5}$$

Taking the derivative of this solution, we finally obtain the solution

$$P_{max}(T) = P_m - P_s \log(T/t_c) \tag{6}$$

for $T \leq t_c$ and

$$P_{max}(T) = P_m - P_l \log(T/t_c) \tag{7}$$

for $T \geq t_c$. This is the result given in Eq. (6).
