# Supplementary material for: A minimal power model for human running performance
Source: PLoS One. 2018 Nov 16;13(11):e0206645. doi: 10.1371/journal.pone.0206645 (PMC6239296; doi:10.1371/journal.pone.0206645)
Supplement: S2 Appendix — (PDF) [file pone.0206645.s002.pdf]

S2 Appendix. Comparison to oxygen uptake measurement

While it is assuring to see below that our model can explain and predict record and individual racing times, a more direct comparison to power output during running is desirable to probe the logarithmic decline of the maximal power output with exercise duration, as predicted by Eq. (6). This is of particular importance in the anaerobic range where different functional forms, e.g., exponential decays, have been proposed [6]. However, running power, as measured by oxygen utilization, can be directly determined only in the aerobic regime. For (supra-maximal) exercise with substantial contributions from anaerobic systems where power output exceeds maximal oxygen uptake, Medbo et al. showed that the oxygen demand can be estimated by extrapolating each runner's individual nominal linear relationship between running speed and submaximal oxygen uptake [1]. The difference between the extrapolated oxygen utilization and the measured oxygen uptake is the accumulated oxygen deficit. Using this method, Medbo et al. determined from treadmill exercise at speeds that caused exhaustion within different predetermined durations the oxygen demand relative to the maximal uptake. Translated to percent of maximal aerobic power output, this oxygen demand is given by $100 \times P_{max}(T)/P_m$ in our model, with $P_{max}(T)$ given in Eq. (6).

While a logarithmic dependence for $P_{max}(T)$ has been deduced from purely empirical data analyses for world records for times above t_c before [6], to our knowledge a logarithmic scaling has not been proposed for shorter exercise with large anaerobic involvement. Hence, it is interesting that there exists experimental estimates of the maximal oxygen utilization that can be maintained for a given duration. As explained above, Medbo et al. [1] obtained for 11 runners data that correspond to $100 \times P_{max}(T)/P_m$ which is shown as function of $T < 5\text{min} \sim t_c$ in Fig. A. We have fitted the prediction of our model to the data, and the results for the runner with smallest and largest oxygen demand are shown in the same figure. The agreement between the data and our model prediction appears to be rather convincing. This suggests that there exists indeed a logarithmic relation between maximally sustainable power and duration in the range of supra-maximal intensities, resembling observation that were made before in the sub-maximal zone.

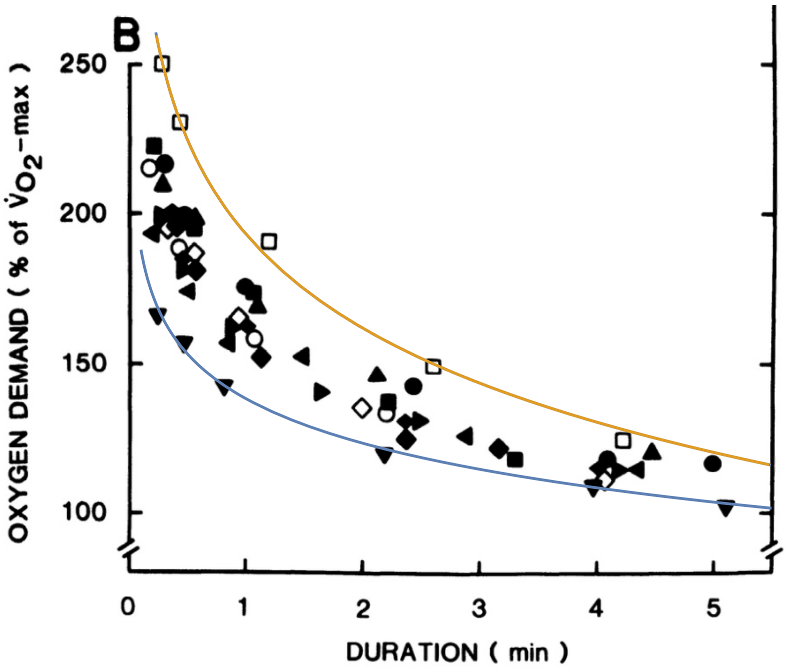

Fig A. Relative nominal oxygen demand as function of the maximum duration over which it can be sustained. Original plot and data for 11 runners from Ref. [1]. The two curves are fits of Eq. (6) to the data for the runners with smallest and largest relative oxygen demand.

References

1. Medbo JI, Mohn AC, Tabata I, Bahr R, Vaage O, Sejersted OM. Anaerobic capacity determined by maximal accumulated O_2 deficit. J Appl Physiol. 1988;64(1):50–60.
